# Supplementary material for: VOC Gas Sensors Based on Zinc Stannate Nanoparticles Decorated with Silver
Source: Nanomaterials (Basel). 2024 Dec 12;14(24):1993. doi: 10.3390/nano14241993 (PMC11728643; doi:10.3390/nano14241993)
Supplement: Supplementary file 1 [file nanomaterials-14-01993-s001.zip › nanomaterials-3306515-supplementary.pdf]

VOC Gas Sensors Based on Zinc Stannate Nanoparticles Decorated with Silver

Svetlana S. Nalimova *, Zamir V. Shomakhov, Dmitry A. Kozodaev, Arina A. Rybina, Sergey S. Buzovkin, Cong D. Bui, Ivan A. Novikov and Vyacheslav A. Moshnikov

NT-MDT BV, 7335 Apeldoorn, The Netherlands; shozamir@yandex.ru (Z.V.S.); kozodaev@ntmdt.nl (D.A.K.); arinasvg02@gmail.com (A.A.R.); sergey.bu2015@gmail.com (S.S.B.); congdoan6997@gmail.com (C.D.B.); i.novikov@ntmdt.nl (I.A.N.); vamoshnikov@mail.ru (V.A.M.)

* Correspondence: s.nalimova@ntmdt.nl

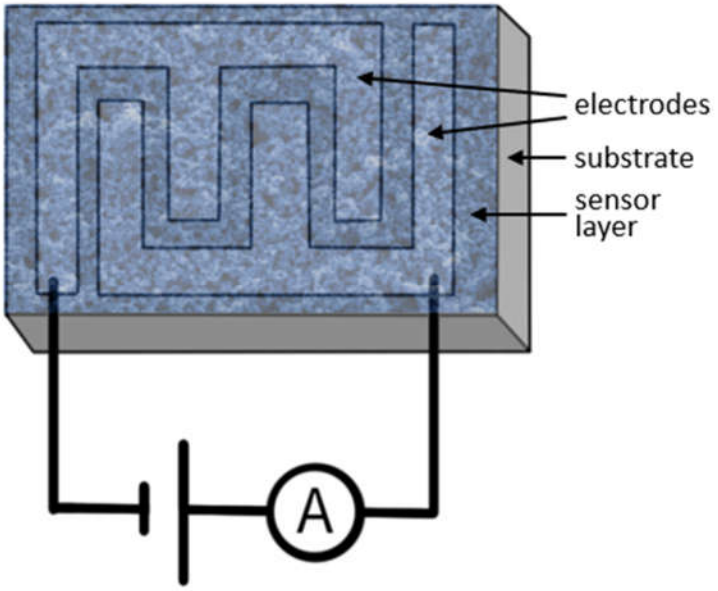

Figure S1. The device scheme.

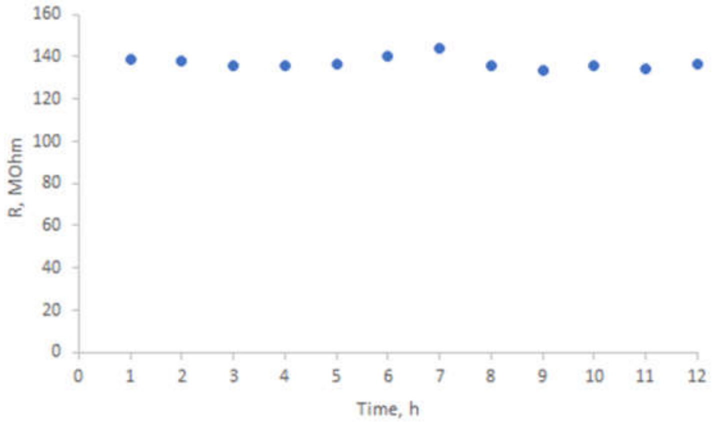

Figure S2. The time dependence of baseline resistance.
